# A Visual and Persuasive Energy Conservation System Based on BIM and IoT Technology

**DOI:** 10.3390/s20010139

**Published:** 2019-12-24

**Authors:** I-Chen Wu, Chi-Chang Liu

**Affiliations:** Department of Civil Engineering, National Kaohsiung University of Science and Technology, Kaohsiung 80778, Taiwan; lucien.chang.tw@gmail.com

**Keywords:** BIM, IoT, persuasive computing, indoor environment comfort level, energy conservation system

## Abstract

Comfort level in the human body is an index that is always difficult to evaluate in a general and objective manner. Therefore, building owners and managers have been known to adjust environmental physical parameters such as temperature, humidity, and air quality based on people’s subjective sensations to yield satisfactory feelings of comfort. Furthermore, electricity consumption could be reduced by minimizing unnecessary use of heating and cooling equipment based on precise knowledge of comfort levels in interior spaces. To achieve the aforementioned objectives, this study undertook the following four tasks: first, providing visualization and smart suggestion functions to assist building managers and users in analyzing and developing plans based on the demands of space usage and electrical equipment; second, using Internet of Things technology to minimize the difference between real situations and those simulated in building information modeling (BIM); third, accurately evaluating interior environment comfort levels and improving equipment operating efficiency based on quantized comfort levels; and fourth, establishing a persuasive workflow for building energy saving systems. Through developing this system, COZyBIM will help to enhance the satisfactions of comfort level in interior space and operate energy consuming equipment efficiently, to reach the target of energy saving.

## 1. Introduction

In recent years, energy consumption of buildings and environmental pollution have become increasingly serious issues, and the concept of the green building has arisen. Following the concept of the green building, the smart building has emerged, which emphasizes more the comfort and convenience of people in the building space. The perception of comfort is majorly influenced by the physical, psychological, and cultural backgrounds and experiences of the individual [1,2]. It is difficult to assess the comfort level of a space in various physical environments when the occupants perform activities indoors in an objective and quantitative manner. Although, in the design planning phase, designers often use stand-alone software such as EnergyPlus [3], Climate Consultant [4], BIM based versions such as Bentley AECOsim Energy Simulator [5], Autodesk Insight 360, and Safaira [6] for building simulation and analysis in operation in space comfort and energy consumption, a major shortcoming of a stand-alone version or a BIM based version of the simulation evaluation software is that it is impossible to simulate the evaluation of the operational phase results on the basis of actual conditions based on the specific space environment. Once the buildings are in operation, they experience unexpected impacting factors, including complex usage of the electrical equipment or space and staff traffic flow. Therefore, some scholars attempt to use IoT (Internet of Things) technology to collect basic environmental information of the locations of individual projects for monitoring the architectural space environment of different projects in a more precise way, even control the environmental equipment via IoT [7]. In this context, recent developments in BEMS are focusing on smart technologies to address the gap between energy consumption and occupants’ comfort [8,9]. Additionally, a multi-dimensional environment also exists that integrates a BIM model as a platform for presenting terminal information and model construction [10,11,12,13] to enhance the visual effect of monitoring results. However, BIM and IoT integration research are still in nascent stages, and data interoperability, interaction, and cloud computing are obvious issues and limitations [14]. To overcome this limitation, this research proposes a visual and persuasive energy conservation system based on BIM and IoT technology. This system not only derives an energy use strategy from the actual experience of the human body, but also facilitates a complete process that can expedite extension of the value and implementation of BIM technology in the later phase of the architectural life cycle. Since the physical quantity of the environment is difficult to discern, the designer or the user often has limited information or even a misunderstanding of the existing environmental state, including simplifications or miscalculations of spatial user behavior patterns, which result in a high degree of inaccuracy in energy performance and related projects, with differences of up to 30% [15,16]. IoT wireless sensing technology is mainly used to gather the large difference in data between simulated results and actual conditions caused by unsuitable climate and environmental data when the building enters its operational phase and to replace past simulating software tools with real physical environmental data. The collected environmental physical data include temperature, humidity, and carbon dioxide concentration in this research, and the comfort analysis of the user’s core role is performed through the calculation module. After establishing the evaluation index of comfort, the visualization methods are used to present the monitored and analyzed comfort level on the user interface. This will enhance user effectiveness and accuracy in the ability of grasping the changes in indoor environment comfort. By means of more accurate planning and forecasting of space use and user behavior patterns, user satisfaction with environmental comfort can be improved and excessive energy usage required for cooling and heating equipment can be reduced.

## 2. Energy Conservation Based on Persuasive Computing

**Figure 1:**
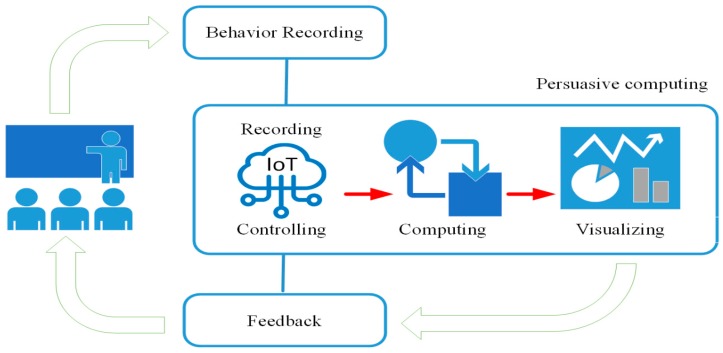
The concept of persuasive computing.

Generally, modern options for energy conservation in buildings are focused on innovative architectures, shapes, structures, materials of a building, and of course systems utilizing renewable energy [17]. However, these methods do not take human comfort according to the different contexts of use into consideration. Barthelmes et al. [18] said that energy efficiency in buildings does not only rely on efficient technical solutions and the design of the building features, but is also highly dependent on how occupants decide to set their comfort criteria, as well as on their energy related and environmental lifestyles. Based on the discussion of existing spatial user behavior patterns, we can define the primary importance of improving space comfort by determining how to improve the user’s objective perception of indoor environmental quality and comfort. Therefore, this research proposed the energy conservation system based on persuasive computing. Persuasive computing, also called ubiquitous computing, is embedding computational capability into day to day objects to make them effectively communicate and perform useful tasks in a way that minimizes the end user’s need to interact with computers as computers [19,20]. Hence, users need a sensory sensing device that uses network communication to store and collect the perceived data for use in a system with visual and interactive interfaces. IoT technology is able to provide these functionalities and applications. The concept of “persuasive” focuses on behavior recording and feedback, then makes incremental, individual behavioral changes. After changing the user’s perception of the environment, the user’s behavior pattern should be changed from the current rigid mode to a more intelligent use process, as shown in Figure 1.

## 3. System Design and Implementation

Energy saving strategies and comfort level will change depending on the type of space. Therefore, this research takes a conference room as the research target. The diverse activities performed in a conference room space may involve a large or small number of users; therefore, the physical environment values are highly explorable. In determining the impact of an indoor space on the comfort of the human body, the most common discrimination item is the indoor environment quality (IEQ), which is a benchmark for residential quality performance. It includes four items: thermal comfort, air quality, noise level, and lighting level [21]. This study focused on two items of the IEQ. One is thermal comfort: when the thermal environment is unsatisfactory, it weakens the “comfort expectation” of other IEQ factors, which accordingly resulting in the less dissatisfaction with other IEQ factors. Moreover, the increase of thermal satisfaction had a positive effect on productivity [22]. Another one is air quality: the level of carbon dioxide in conference rooms has a significant effect on the comfort of the working conditions [23,24]. As long as the indoor space has more than one user, the physical quantity of the two indoor items of thermal physical quantity and air quality (carbon dioxide output) would not disappear due to temporary variation in space usage. Besides, this research excluded two IEQ items: noise level and lighting level are characterized by the inability to be homogenized and objectively assessed due to changes in usage conditions. For example, consider the noise and lighting levels in the conference room: when the conference room users have a group meeting, the volume generated may be very different from that when a speaker simply is presenting. Moreover, the ambient light source needs to be reduced during a slide presentation, which differs in the degree of illumination in a static conference room [25]. Therefore, this study establishes a visual and persuasive energy conservation system, namely COZyBIM, which can evaluate and visualize the comfort of indoor spaces in order to provide a stable and comfortable environment for users of a space for a long time.

### 3.1. System Framework

**Figure 2:**
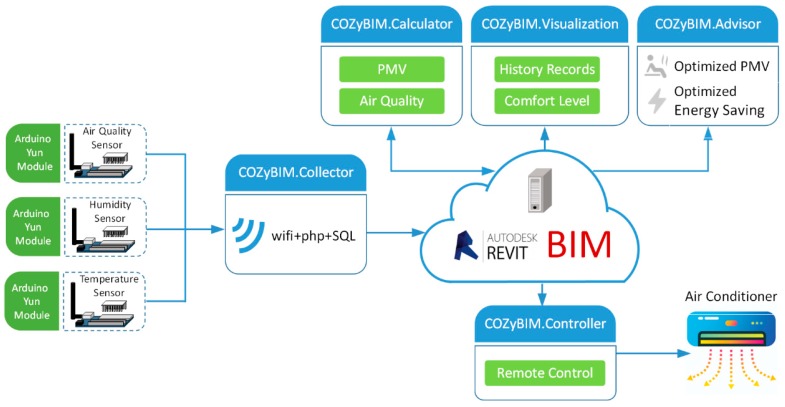
COZyBIM system architecture diagram.

This research institute mainly focused on “persuasive energy conservation” as the core design concept. To achieve this goal, the system focused on system design and implementation of six key points: data perception, information transmission and storage, visual result presentation, automatic control, and energy use strategy recommendations. It is a reasonable behavior that upon entering the space, the user switches on the equipment to the operating mode; however, during a three hour course, when the quality of the closed environment deteriorates, the user must actively respond to and improve the comfort of the space. Therefore, the sensing module, the computing module, and the visualization module of the system can be used to determine the instantaneous state of environmental comfort and, finally, according to the setting suggestion of the device provided by the persuasive energy saving suggestion module; at the digital desk, the switch or a set values of the air-conditioner and other related electrical equipment (such as a cooling fan) is adjusted through the control module. The system architecture diagram is shown in Figure 2. The details of each module are described in the following sections.

### 3.2. Graphical User Interface

In the absence of a central management system configuration, the instructor or a teaching assistant must act as a space manager during the course. In addition, because the system is in operation during the teaching, the interface of the system needs to have intuitive data presentation and simple operation rules to reduce the time spent by the user on system operation. Therefore, this study develops system interface design points suitable for the purpose of “indoor space comfort analysis”, “environmental real-time monitoring and equipment control”, and “persuasive energy conservation and strategy analysis” and follows the following interface design principles:

- 2D and 3D data are combined to provide users with a more complete decision making reference.
- Quickly enables common features.
- Distinguishes between primary and secondary function areas, avoiding unnecessary functional components from being displayed on the system interface.

### 3.3. Smart BIM

All information delivery architectures use Smart BIM modules as the core information platform, such as the core processor and brain of the system, dedicated to information integration, analysis, calculation, presentation, and command delivery. The information collected by the front-end sensing module will be transmitted to the Seeed Studio Wio Node’s user database via WiFi. The user can access it through the URL and the sensing module’s ID name in the web browser. This study uses the functional window program for data capture provided by Newtonsoft.Json. At the same time, the captured data are stored in the Smart BIM integrated data model set up in this study. Subsequently, data retrieval, analysis, and visualization are performed. This study uses Autodesk Revit as the BIM model to build and integrate with the COZyBIM system. It is mainly for realizing the integrated user process; the functions of model construction, analysis operation, decision support, and automatic control can operate in the same software interface, thereby avoiding inconvenience and risk of cross-interface. The Smart BIM module has integrated and visual features, and the environment will be a three-dimensional building information model that originally contains object attributes and geographic information, giving information to another dimension, real-time physical environment status. In addition, when the original BIM model is linked to an external database, it has the ability to reflect real-time conditions. The user can adjust the usage strategy according to the visual result, that is the BIM model has been successfully intelligentized as an important tool for re-analysis and design in the operation and maintenance phase.

### 3.4. The Five Kernel Module

(1) COZyBIM.Collector

**Figure 3:**
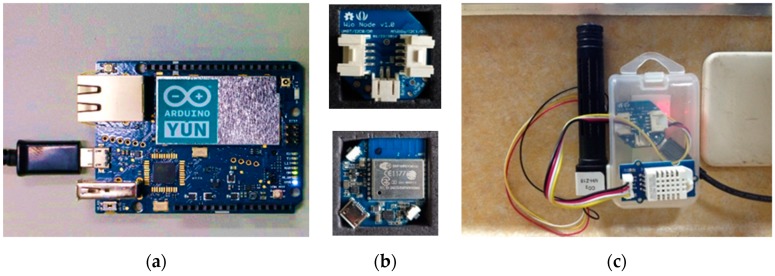
Actual installation status of the sensor. (**a**) Arduino Yun; (**b**) Wio Node; (**c**) Sensor Assembly.

The IoT sensing module used in the COZyBIM system is the processor of Arduino Yun and Seeed Studio Wio Node, and it uses a DHT22 temperature and humidity sensor and a carbon dioxide concentration sensor as sensing components. The actual installation status is shown in Figure 3. The sensor ID can be used anytime and anywhere, and the sensing data can be retrieved any time. The sensing data are accessed and collected every 15 min.

(2) COZyBIM.Calculator

The thermal comfort standard considers the satisfaction of people within a standard environment for evaluating the thermal environment, rather than in a certain stable indoor situation. The most widely used thermal comfort indicator in the world is the PMV model proposed by Fanger [26]. Therefore, the comfort level calculation module uses the formula of PMV calculation. This module extracts the temperature and humidity data from the system, which are converted and calculated by the formula proposed by Fanger to obtain the PMV value as shown in Equation (1). Note that the metabolic rate (M) and the clothing insulation (I_cl_) cannot be calculated by any mathematical function. Therefore, it must be set in the user interface according to the user experience and the actual situation on site. The metabolic rate of the human body and the amount of clothing insulation can be referenced by the set values in Table 1 and Table 2, and the remaining parameters that can be converted are shown in Table 3.

PMV = (0.303e^−0.0036M^ + 0.028) × {M − 3.05 × 10^−3^ × (5733 − 6.99M − P_a_) − 0.42 × (M − 58.15) − 1.7 × 10^−5^ × M × (5867 − P_a_) − 0.0014 × M × (34 − t_a_) − 3.96 × 10^−8^f_cl_ × [(t_cl_ + 273)^4^ − (t_r_ + 273)^4^] − f_cl_ × h_c_ × (t_cl_ − t_a_)} (1)

In terms of air quality, the most significant source of air pollution in an indoor environment is carbon dioxide [27]. Excessive use of the air-conditioning system may negatively impact the indoor air quality, which is often overlooked by users. Both Taiwan and the U.S. Environmental Protection Agency recommend 1000 ppm [28,29,30]. Therefore, when the concentration of carbon dioxide in the indoor space increases to a certain limit, the system should warn the indoor user to introduce outdoor air to reduce the indoor carbon dioxide concentration and reduce the discomfort of the indoor space user. According to the abovementioned specifications, this study uses 1000 ppm as the standard limit for indoor carbon dioxide concentration.

(3) COZyBIM.Visualization

**Figure 4:**
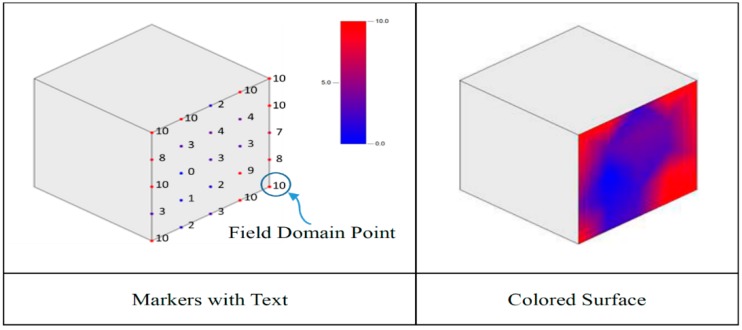
Revit analysis display style category function diagram.

In the visualization module, the Autodesk Revit API (application programming interface) is used to develop the visual display function of the data, and the environmental comfort calculation result data are fed back to the BIM model to provide effective information for the building management personnel in order to perform environmental control and decision analysis. First, an analysis result container must be built through the API, in which the user defined field domain points and field values can be loaded. In the test environment of this study, the positions of the four sets of sensing modules are used as the relative positions of the regional points; each module is then transmitted back to the calculation module, and the calculated PMV value is fed back to the field values. In the visualization module, the color setting can be performed for the max and min color. All the area point values will be defined according to the color of the numerical boundary, showing a gradient color visualization. The effect is shown in Figure 4.

COZyBIM.Visualization aims to provide users with the opportunity to have custom boundary conditions for the space that is analyzed, so users can customize the different areas in a single large space to define the scope for visual analysis. In this way, the system can have greater flexibility and efficiency to provide important information to users.

(4) COZyBIM.Advisor

ISO 7730’s [31] definition of thermal comfort is that when the PMV is between −0.5 and 0.5, it is considered within the comfortable range, but due to people’s physiological differences, 10% of people still feel unsatisfied. The ASHRAE 55 [32] also calculates the comfortable temperature range on the basis of PMV. Therefore, the persuasive energy saving suggestion module according to the abovementioned range provides air-conditioning temperature setting recommendations. It mainly provides two options: One is that “based on the optimization of PMV comfort”, the existing indoor space temperature is used as a calculation reference, and the result is deemed the best when the PMV value approaches zero. When the PMV value is close to zero, it is possible to know the indoor temperature that can satisfy the PMV value optimization in the existing space state, which is the recommended value for setting the air conditioner temperature. The second is “the most energy saving effect based on the comfort range”, which is the maximum value of the PMV comfort range −0.5 to +0.5 as the result of the calculation. The higher the PMV value, the greater the temperature will be proportional to the PMV. When the temperature is higher, the time and power consumption of the cooling compressor will be reduced. Therefore, in this study, the PMV value of +0.5 is used as the recommended condition for air-conditioning temperature setting. Further, calculation is performed to obtain a recommended value that is sufficient for an appropriate comfort level and maximum power saving.

(5) COZyBIM.Controller

To increase the system integrity of COZyBIM, users can control and adjust the equipment in the real space directly in the Smart BIM user interface after receiving the spatial comfort analysis result and the recommended values for the air-conditioning temperature setting for improving management efficiency and convenience. Therefore, after the user selects the temperature of the air conditioner to be set through the visual interface, the system directly controls the air-conditioner temperature setting by transmitting the frequency to the infrared emitter of the air-conditioner of the actual space through the URL protocol.

## 4. The Experiment

**Figure 5:**
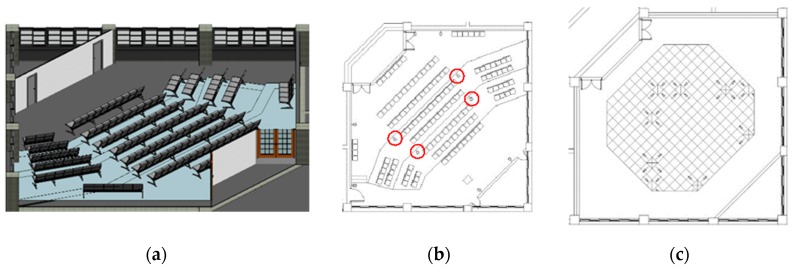
The BIM model and space configuration. (**a**) BIM Model; (**b**) Sensor Setup Location; (**c**) Air Conditioning Outlet.

This study selected the large 702 conference room of the Department of Civil Engineering of Kaohsiung University of Science and Technology as the site for the case analysis and system function testing. The conference room is the largest public meeting space and classroom in the Department of Civil Engineering and can accommodate up to 130 people. The space is fan-shaped and has an indoor configuration that gradually stacks up from front to back. The conference room is used for diverse purposes and not just as a general classroom. In addition, lectures and student activities are also held in the conference room. The BIM model and space configuration are shown in Figure 5.

**Figure 6:**
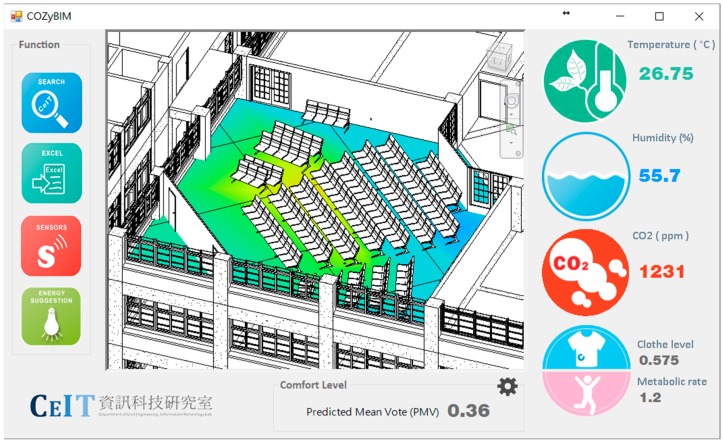
Graphical user interface of COZyBIM.

The conference room is located on the top floor of the building. In addition to the heat radiation from the sides, it also receives heat radiation from the top floor. Therefore, during normal times, direct sunlight through the windows on both sides is blocked using curtains, which in turn leads to sultry indoor conditions. Space users’ environmental perceptions and corresponding behaviors are quite limited. The only method to increase ventilation and reduce the indoor temperature is to turn on the air-conditioner. The air-conditioner is simply turned on without managing the temperature. Due to the lack of a sensing and monitoring control system for indoor environments, students and instructors are often subjected to increased carbon dioxide concentrations and too low air-conditioning temperature, resulting in deterioration of indoor environmental quality and excessive energy use. The graphical user interface of COZyBIM designed according to the above principles is shown in Figure 6.

**Figure 7:**
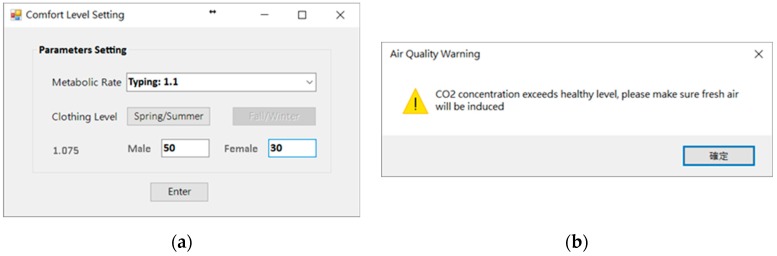
Setting and warning window of COZyBIM. (**a**) Activity Status and Clothing Setting Screen for Space Users; (**b**) Carbon Dioxide Concentration Detection Warning Window.

**Figure 8:**
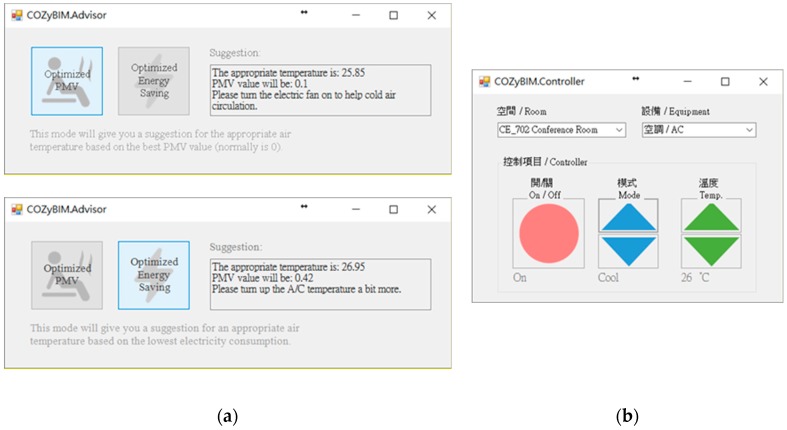
Advisor window and control window of COZyBIM. (**a**) Inductive Energy-Saving Module Function; (**b**) Smart Control Module Function Window.

**Figure 9:**
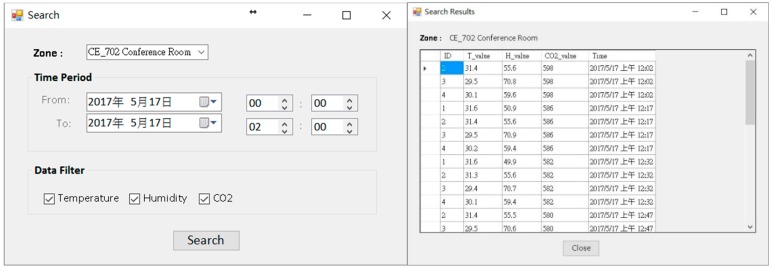
Historical data search function.

**Figure 10:**
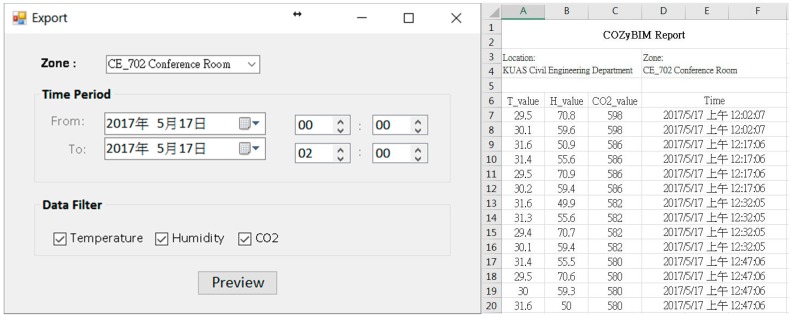
Data exported to the excel format file function.

For the demonstration, the environmental data of 17 May 2016 were used as an example of the system function display. The usage period of the day was afternoon, and the number of people was about 80. The type of activity was sitting and writing activities. The degree of clothing was based on the latest weather conditions and actual conditions. Users wore short-sleeved tops and pants (see Figure 7a). At the start of the use of the space, the system user, who was also the space manager, opened the COZyBIM user interface to observe the distribution of thermal comfort and the instantaneous data of carbon dioxide concentration to determine whether adjustments needed to be made to the air-conditioner or other auxiliary equipment to improve on-site comfort conditions. If the carbon dioxide concentration when the COZyBIM system was under operation exceeded the limit of 1000 ppm, the system would display a warning window. As shown in Figure 7b, the user would be reminded to open the door or window or use other methods to introduce external air to reduce indoor carbon dioxide concentration. The environmental information and PMV value would be displayed in the user interface (As shown in Figure 6). At 15:42 (use time was 13:30 to 16:20), the average indoor temperature was 27.75 °C, humidity was 55.7%, carbon dioxide concentration was 1231 ppm, and the PMV value was 0.36. It can be seen that the indoor thermal comfort level at that time was in the range of −0.5 to +0.5, while the carbon dioxide concentration was in an excessive state and in the range of the harmful level. In this case, we could still use the recommended function of the inductive energy saving module to obtain the most comfortable temperature for the current site conditions (as shown in Figure 8a). During this time period, the indoor temperature that best satisfied the best PMV value was 25.85 °C. At this temperature and the rest of the parameters such as the activity status of the space user, the degree of clothing, and humidity, the PMV value would drop to 0.1. According to the definition of ISO 7730, under this PMV value, nearly 95% of indoor space users would be satisfied with the comfort of the thermal environment. If the recommended value of the air-conditioner was required to achieve the energy saving target, the function of “Optimized Energy Saving” could be selected. After the selection, the system met the limit of the PMV comfort range +0.5 for 90% of the indoor users as the end point of the loop analysis. The obtained temperature was 26.95 °C, and the PMV value was 0.42. When the system user obtained warning information and temperature control suggestions from the user interface of COZyBIM, in addition to facilitating ventilation to reduce the concentration of carbon dioxide, through the intelligent controller of COZyBIM (as shown in Figure 8b), the user can adjust the switch, mode, and set values of the energy consuming device. In addition, users can also access the environmental data of different periods and automatically generate in Excel format the data through the functions of searching and exporting into an Excel spreadsheet. For other analyses, the data included are temperature, humidity, carbon dioxide concentration, and data storage time. The results are shown in Figure 9 and Figure 10.

## 5. Discussion

**Figure 11:**
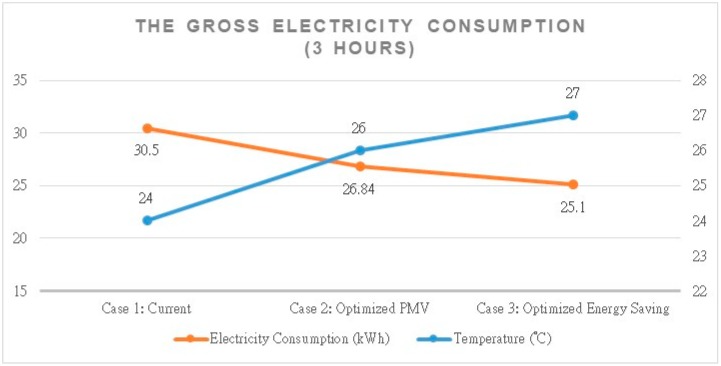
The different settings’ analysis and comparison for energy conservation.

The space manger can monitor the class situation and control the equipment automatically by the COZyBIM system. The results of energy conservation after using the system are shown in Figure 11. Case 1 was the previous condition without using the proposed system. It was measured by an electric meter, and the result of the measurement was 30.50 kWh during three hours. Many scholars have researched the relationship between temperature and energy saving. According to the results of Roussac’s research [33], the one degree temperature setpoint increase produced a 6% reduction in daily HVAC electricity consumption. Case 2 was using the recommend value about optimized PMV, and the gross electricity consumption was 26.84 kWh during three hours. Case 3 was using the mode of optimized energy saving, and the gross electricity consumption would become 25.10 kWh. The different temperature settings provided by the COZyBIM system could generate different efficiencies for energy saving.

## 6. Conclusions

This study used IoT wireless sensing technology to collect physical data of a real-world environment, and with the parameterization and visualization functions of the proposed BIM multi-dimensional model, the data related to environmental comfort level were added and presented using graphs and tables in the user interface. Using this system, the gap between virtual models and real space could be reduced, so that system users and space managers had more reference paths and tools to improve the operational efficiency and objective comfort perception of the space, space users, and heating and cooling equipment.

In terms of the effectiveness of the monitoring results, through visual means, the system user could intuitively judge whether the current thermal comfort level of the space met objective quantitative standards. The user could also determine whether the carbon dioxide concentration was at or near the recommended limit during the time of activity. If the level exceeded the limit, the monitoring result displayed a warning that the existing air-conditioning equipment was not maintaining a balance of the indoor air quality. As a result, space users must be active in the state of excessive carbon dioxide concentration for a long time. Therefore, this study suggested that when carrying out activities in a large indoor public meeting space, it is still necessary to install a ventilator or moderately introduce air from the outside to help exchange indoor and outdoor air to reduce the amount of carbon dioxide accumulated indoors.

In terms of thermal comfort level, with the use of intelligent decision making functions, this study found that when the indoor temperature dropped to 26–27.5 °C, the PMV indicator could enter the comfort range of −0.5 to +0.5. These data could serve as a reference for space managers to replace future air-conditioning equipment.

This study proposed a feasible BIM based indoor environment monitoring and comfort analysis system and its construction process. The system had the advantages of low cost and easy equipment acquisition. In conjunction with the function of the comfort level calculation module, the feedback reference for the device use was given to achieve the purpose of energy saving. That is, it was explicitly recommended that the space manager or system user set the value of the heating and cooling equipment to meet appropriate thermal comfort levels under the current conditions. If the manager of the space could refer to the advice given by the COZyBIM system, he/she could save energy, improve the efficiency of the equipment, and maintain the comfort for the space user.

In the future, this system will include more data monitoring functions, as well as over-voltage sensors and relay devices. We can then monitor the real-time power consumption of the electrical equipment and can also use remote control methods to adjust or switch on and off the electrical equipment. Together with analysis results for IEQ, we can not only work in a comfortable environment, but also enhance the efficiency of electrical equipment.

## Figures and Tables

**Table 1:** Human metabolism quantitative reference [26].

Type of Human Activity	M	
	W/m^2^	MET
Lying flat	46	0.8
Sitting down	58	1.0
Sitting position for desktop activities	70	1.2
Standing with minor activity	93	1.6
Standing with moderate activity	116	2.0

**Table 2:** Clothing insulation numerical reference [26].

Everyday Wear	I_cl_	
	m^2^K/W	clo
Short shirt, T-shirt, shirt, thin socks, sandals	0.050	0.3
Lined pants, short sleeved shirts, light trousers, thin socks, shoes	0.080	0.5
Shorts, slips, pantyhose, skirts, shoes	0.105	0.7
Underwear, shirts, trouser, socks, shoes	0.110	0.7

**Table 3:** Source of the parameters of PMV.

Parameter	Parameter Source
t_a_ dry bulb/air temperature (°C)	Collected directly by DHT-22 temperature and humidity sensor
P_a_ partial vapor pressure	The relative humidity collected by the DHT-22 temperature and humidity sensor and converted by the formula
V_ar_ wind speed (m/s)	With a minimum breeze volume of 0.1 m/s as an indoor preset value
t_cl_ clothing surface temperature (°C)	Converted by calculation
t_r_ black ball temperature/average radiant temperature (°C)	Equivalent to t_a_ dry bulb temperature
h_c_ convective heat exchange coefficient (W·m^−2^·K^−1^)	Converted by calculation
f_cl_ clothing surface area factor	Converted by calculation

## References

[1] Van Hoof J., Schellen L., Soebarto V., Wong J.K.W., Kazak J.K. (2017). Ten questions concerning thermal comfort and ageing. Build. Environ..

[2] Yang L., Yan H., Lam J.C. (2017). Thermal comfort and building energy consumption implications—A review. Appl. Energy.

[3] Shabunko V., Lim C.N., Mathew S. (2018). EnergyPlus models for the benchmarking of residential buildings in Brunei Darussalam. Energy Build..

[4] Milne M., Liggett R., Benson A., Bhattacharya Y. Climate Consultant 4.0 Develops Design Guidelines for Each Unique Climate. Proceedings of the American Solar Energy Society.

[5] Šekularac N.D., Šumarac D.M., Tovarović-Cikic J.L., Čokić M.M., Ivanović-Šekularac J.A. (2018). Re-use of historic buildings and energy refurbishment analysis via building performance simulation a case study. Therm. Sci..

[6] Al-Saeed Y.W., Ahmed A. (2018). Evaluating Design Strategies for Nearly Zero Energy Buildings in the Middle East and North Africa Regions. Designs.

[7] Jia M., Komeily A., Wnag Y., Srinivasan R.S. (2019). Adopting Internet of Things for the development of smart buildings: A review of enabling technologies and applications. Autom. Contr..

[8] Gómez-Romero J., Molina-Solana M., Ros M., Ruiz M.D., Martin-Bautista M.J. (2018). Comfort as a service: A new paradigm for residential environmental quality control. Sustainability.

[9] Boodi A., Beddiar K., Benamour M., Amirat Y., Benbouzid M. (2018). Intelligent Systems for Building Energy and Occupant Comfort Optimization: A State of the Art Review and Recommendations. Energies.

[10] Wang H., Gluhak A., Meissner S., Tafazolli R. Integration of BIM and live sensing information to monitor building energy performance. Proceedings of the 30th CIB W78 International Conference.

[11] Chen J., Bulbul T., Taylor J.E., Olgun G. A case study of embedding real-time infrastructure sensor data to BIM. Proceedings of the Construction Research Congress.

[12] Marzouk M., Abdelaty A. (2014). Monitoring thermal comfort in subways using building information modeling. Energy Build..

[13] Nguyen H.T. (2016). Integration of BIM and IoT to Improve Building Performance for Occupants’ Perspective. Master’s Thesis.

[14] Tang S., Shelden D.R., Eastman C.M., Pishdad-Bozorgi P., Gao X. (2019). A review of building information modeling (BIM) and the internet of things (IoT) devices integration: Present status and future trends. Autom. Contr..

[15] GhaffarianHoseini A., Zhang T., Nwadigo O., GhaffarianHoseini A., Naismith N., Tookey J., Raahemifar K. (2017). Application of nD BIM Integrated Knowledge-based Building Management System (BIM-IKBMS) for inspecting post-construction energy efficiency. Renew. Sust. Energy Rev..

[16] Hong T., Taylor-Lange S.C., D’Oca S., Yan D., Corgnati S.P. (2016). Advances in research and applications of energy-related occupant behavior in buildings. Energy Build..

[17] Chwieduk D.A. (2017). Towards modern options of energy conservation in buildings. Renew. Energy.

[18] Barthelmes V., Fabi V., Corgnati S., Serra V. (2019). Human Factor and Energy Efficiency in Buildings: Motivating End-Users Behavioural Change. Adv. Intel. Syst. Comput..

[19] Van’t Hooft M., Swan K. (2006). Ubiquitous Computing in Education: Invisible Technology, Visible Impact.

[20] Oinas-Kukkonen H., Harjumaa M. (2009). Persuasive Systems Design: Key Issues, Process Model, and System Features. CAIS.

[21] Lee M.C., Mui K.W., Wong L.T., Chan W.Y., Lee E.W.M., Cheung C.T. (2012). Student learning performance and indoor environmental quality (IEQ) in air-conditioned university teaching rooms. Build. Environ..

[22] Geng Y., Ji W., Lin B., Zhu Y. (2017). The impact of thermal environment on occupant IEQ perception and productivity. Build. Environ..

[23] Tham K.W. (2016). Indoor air quality and its effects on humans—A review of challenges and developments in the last 30 years. Energy Build..

[24] Teleszewski T., Gładyszewska-Fiedoruk K. (2019). The concentration of carbon dioxide in conference rooms: A simplified model and experimental verification. Int. J. Env. Sci. Technol..

[25] Nagy Z., Yong F.Y., Frei M., Schlueter A. (2015). Occupant centered lighting control for comfort and energy efficient building operation. Energy Build..

[26] Fanger P.O. (1970). Thermal Comfort, Analysis and Applications in Environmental Engineering.

[27] Seppänen O.A., Fisk W.J., Mendell M.J. (1999). Association of ventilation rates and CO_2_ concentrations with health and other responses in commercial and institutional buildings. Indoor Air.

[28] Wargocki P., Da Silva N.A.F. (2015). Use of visual CO_2_ feedback as a retrofit solution for improving classroom air quality. Indoor Air.

[29] CEN-EN 13779 (2007). Ventilation for Non-Residential Buildings-Performance Requirements for Ventilation and Room-Conditioning Systems.

[30] Satish U., Mendell M.J., Shekhar K., Hotchi T., Sullivan D., Streufert S., Fisk W. (2012). Is CO_2_ an indoor pollutant? Direct effects of low-to-moderate CO_2_ concentrations on human decision-making performance. Env. Health Persp..

[31] ISO 7730 (2005). Ergonomics of the Thermal Environment-Analytical Determination and Interpretation of Thermal Comfort Using Calculation of the Pmv and Ppd Indices and Local Thermal Comfort Criteria.

[32] ASHRAE Standard 55 (2017). Thermal Environmental Conditions for Human Occupancy.

[33] Roussac A.C., Steinfeld J., de Dear R. (2011). A preliminary evaluation of two strategies for raising indoor air temperature setpoints in office buildings. Archit. Sci. Rev..

